# A Case of Hyperammonemia Not Attributable to Liver Disease and Treated With IV Ammonia Scavengers

**DOI:** 10.7759/cureus.74028

**Published:** 2024-11-19

**Authors:** Joel Thomas, Astly George, Sharmin Mrittika, Bilal Ahmad, Gisela Wilcox

**Affiliations:** 1 Department of Internal Medicine, Wrexham Maelor Hospital, Wrexham, GBR; 2 Department of Gastroenterology, Wrexham Maelor Hospital, Wrexham, GBR

**Keywords:** ammonia scavengers, arginine, non-cirrhotic hyperammonemia, ornithine transcarbamylase deficiency, orotic acid, phenylbutyrate, plasma amino acids, sodium benzoate, type ii citrullinemia, urea cycle disorder

## Abstract

Hyperammonemia is a serious metabolic condition marked by elevated ammonia levels in the blood, leading to neurological damage and systemic complications if untreated. While often associated with liver dysfunction, inborn metabolic errors such as fatty acid oxidation defects, pyruvate metabolism disorders, urea cycle disorders (UCDs), urea splitting bacterial infections, hemato-oncological disorders, and portosystemic shunts are less commonly recognized but significant causes, particularly outside neonatal populations. These metabolic errors, due to partial enzyme deficiencies, may present later in life with atypical symptoms.

We report an acute presentation of a female patient in her late fifties with a background of noncirrhotic hyperammonemia of unknown etiology, controlled with oral sodium benzoate. She presented with ataxia, altered mental status, and delusion. The laboratory evaluation revealed significantly elevated ammonia levels, which did not respond to an increased dose of oral sodium benzoate, and she required intravenous ammonia scavengers to achieve acceptable levels. We further discuss several investigations done to establish a cause for her hyperammonemia and a psychiatric diagnosis of erotomania/de Clerambault’s syndrome secondary to recurrent hyperammonemia. Although her biochemical workup had some features suggestive of type 2 citrulline deficiency, SLC25A13 mutation analysis for citrin deficiency and an extended R98 panel were negative. Thus, highlighting the complexity of diagnosis of inborn metabolic errors and treatment of metabolic hyperammonemia in the absence of an established diagnosis.

It also emphasizes the need for heightened awareness and prompt treatment of inborn metabolic errors in adult patients, following the British Inherited Metabolic Disease Group (BIMDG) management guidelines to prevent severe neurological outcomes. Multidisciplinary management, including liaison with specialists in metabolics, gastroenterology, and dietetics, is crucial for optimizing patient care and outcomes in such complex cases.

## Introduction

In 90% of instances, hyperammonemia is associated with severe liver illness; the remaining 10% is caused by other disorders that interfere with its production or excretion [1]. For hyperammonemic patients without significant liver disease, less common noncirrhotic causes should also be evaluated, including potential late-onset inborn errors of metabolism (IEM) such as fatty acid oxidation disorders, pyruvate metabolism abnormalities, urea cycle disorders (UCDs), and drug effects, particularly from medications like valproate. IEMs are commonly found in pediatric and neonatal populations. Confusion or neurological symptoms like seizures are among the early signs of noncirrhotic hyperammonemic encephalopathy (NCHE), which can progress to potentially fatal cerebral swelling and herniation [2]. Symptoms may appear following catabolic stressors such as disease, pregnancy, or an increase in protein intake. Additionally, recent research suggests a potential link between patients with inherited metabolic disorders (IEMs) developing hepatic dysfunction, underscoring the need for heightened clinical vigilance in patients with unexplained hyperammonemia [3]. Appropriate and timely management requires a solid understanding of the fundamental pathophysiology, differential diagnosis, and treatment approaches available.

## Case presentation

A 58-year-old female presented with progressive worsening of confusion and unsteadiness on her feet for three days. She gave no history of fever, cough, nausea, vomiting, abdominal pain, headache, neck stiffness, adherence to a vegetarian diet, or alcohol. She is known to have had these symptoms secondary to hyperammonaemia in the past. Her family reported that she has had a delusional belief for the past six months that she is in a relationship with a famous guitarist. Acting on this belief, she traveled abroad to meet the individual, requiring her family to involve the police to arrange her return. Although she is known to have had mild depression in the past, she had no other psychiatric illnesses.

She first presented with ataxia and confusion at another hospital, where elevated ammonia levels (>100 µmol/L) were detected four years ago. She underwent various tests, including CT scans of the head, abdomen-pelvis, MRI of the brain, and EEG, all of which were inconclusive except for fatty liver on CT. It was then suggested that an underlying inborn error of metabolism might be responsible, and she was referred to the regional metabolic team to investigate. Under their care, her ABG showed metabolic alkalosis with a normal anion gap and normoglycemia. They proceeded to perform an analysis of her plasma amino acid levels, which are detailed in Table 1.

**Table 1:** Plasma amino acids level

Plasma amino acid	Report	Normal range
Citrulline	70	10-40 umol/L
Arginine	118	12-145 umol/L
Taurine	55	28-212 umol/L
Valine	120	77-335 umol/L
Aspartic acid	12	4-52 umol/L
Cystine	7	10-80 umol/L
Hydroxyproline	16	6-75 umol/L
Methionine	42	11-43 umol/L
Threonine	169	47-228 umol/L
Isoleucine	39	27-101 umol/L
Serine	79	66-231 umol/L
Leucine	81	52-206 umol/L
Asparagine	44	23-80 umol/L
Tyrosine	151	32-125 umol/L
Glutamic acid	166	21-194 umol/L
Phenylalanine	93	35-103 umol/L
Glutamine	629	227-735 umol/L
Ornithine	92	24-144 umol/L
Proline	276	98-429 umol/L
Lysine	177	70-259 umol/L
Glycine	321	133-455 umol/L
Histidine	77	42-108 umol/L
Alanine	389	138-565 umol/L
Tryptophan	38	19-107 umol/L

The results showed a plasma citrulline of 70 µmol/L (normal: 10-40), elevated tyrosine at 151 µmol/L, and arginine at 118 µmol/L. The threonine-to-serine ratio was 2.14 (normal: <1.0). In addition to this, her urine tested negative for arginosuccinate and orotic acids. Although these findings were not severely elevated, the possibility of a diagnosis of type 2 citrullinemia or citrin deficiency was considered.

Given the possibility of citrin deficiency, under the guidance of the metabolic team, the patient was started on sodium benzoate 500 mg TDS, and a DNA test to detect SLC25A13 mutation was requested. She responded well to the treatment, her ammonia levels were well-controlled, and she remained asymptomatic. Thusforth, the patient was regularly followed up in metabolic and gastroenterology clinics while awaiting her DNA test report. A year later, her ultrasound still revealed non-alcoholic fatty liver disease (NAFLD), but it progressed to cirrhosis within another year. An esophagogastroduodenoscopy (OGD) revealed grade 1 varices and a liver biopsy confirmed cirrhosis, consistent with non-alcoholic steatohepatitis (NASH). Unfortunately, the pathology of NASH cirrhosis is indistinguishable from citrullinemia-related cirrhosis. Due to the presence of cirrhosis and its potential contribution to hyperammonemia, the patient was started on Rifaximin and Lactulose.

Despite this, she had two additional admissions, during which her sodium benzoate dosage was increased to 1 g TDS. She responded positively to these adjustments and was discharged with dietary recommendations. Her response was reassuring, as hyperammonemia primarily due to liver disease typically does not respond to benzoate therapy.

At this stage, the patient relocated to our area and was transferred to our care. Her SLC25A13 gene mutation test came back negative, so we sent another sample to test an extensive range of genes, including the R98 panel for inborn metabolic errors known to cause hyperammonemia and alpha-1-antitrypsin to rule out its deficiency. The R98 panel uses whole genome sequencing to screen for mutations of *ACADM, ACADVL, ALDH18A1, ARG1, ASLL, ASS1, AUH, BCKDHA, BCKDHB, CA5A, CPS1, CPT1A, CPT2, DBT, ETFA, ETFB, ETFDH, GLUD1, HADHA, HADHB, HLCS, HMGCL, IVD, MLYCD, MMAA, MMAB, MUT, NAGS, OAT, OTC, PC, PCCA, PCCB, POLG, PYGM, SERAC1, SLC22A5, SLC25A13, SLC25A15, SLC25A20, SLC7A7, *and *TMEM70*. These include urea cycle defects, fatty acid oxidation disorders, and amino acid metabolism disorders. Meanwhile, we also did an acylcarnitine blood spot test, which did not detect any evidence of fatty acid oxidation disorder.

Her other medical history includes type 2 diabetes mellitus (T2DM), chronic obstructive pulmonary disease (COPD), osteoporosis, and vitamin B12 deficiency. She has a notable family history, including the early death of her mother and a sister who passed away in infancy due to an unknown disease. Her daughter had delayed motor milestones and only started walking at age six. The patient herself experienced developmental delays in motor and speech skills during childhood.

During her current presentation with confusion, delusional belief, and ataxia, the general examination revealed that she was pale, exhibited an ataxic gait, had bilateral finger clubbing, and showed flapping tremors. Although the neurological examination did not identify any specific deficits, she tested positive for Romberg's sign (most probably secondary to diabetic neuropathy). Given her extensive medical history, alongside routine blood tests, serum ammonia, plasma amino acids, urine amino acids, and blood spot acylcarnitine were requested (Table 2). 

**Table 2:** Blood tests on admission WBC: white blood cells, PT: prothrombin time, APTT: activated partial thromboplastin time, eGFR: estimated glomerular filtration rate, CRP: c-reactive protein

Test	Result	Normal range
Ammonia	153	<50
WBC	10.6	4-11x10^9^ /L
Hemoglobin	148	115-165 g/L
Platelets	138	150-400x10^9^/ L
PT	11.4	10-14 sec
APTT	26.5	21-35 sec
Fibrinogen	3.5	2-4 g/L
Glucose	7.7	4.4-7.8 mmol/L
Bilirubin	23	<21 umol/L
Protein	66	60-80g/L
Albumin	37	35-50g/L
Alkaline phosphatase	169	30-130U/L
Alanine transaminase	23	<33U/L
Sodium	140	133-146 mmol/L
Potassium	3.3	3.5-5.3 mmol/L
Urea	3.8	2.5-7.8 mmol/L
Creatinine	76	53-97.2 umol/L
eGFR	68	>90 ml/min/1.73m^2^
CRP	5	<5 mg/L

Her urine was positive for glycine secondary to benzoate therapy, while urine organic acids and blood spot acylcarnitine were negative. She was promptly started on an increased dose of sodium benzoate at 2 g TDS, and her lactulose dose was raised to 30 mL TDS on day 1 while maintaining the same Rifaximin dosage of 550 mg BD. An MRI of the brain was performed to assess her confusion and check for signs of hyperammonemic encephalopathy, and it revealed moderate periventricular chronic ischemic changes. An abdominal ultrasound was conducted to evaluate portal flow and the progression of cirrhosis, which suggested no significant changes. At this point, her R98 panel identified no pathogenic variants in any of the genes screened, and alpha-1 antitrypsin was sufficient. A genetic diagnosis could not be entirely ruled out as the test does not cover the mitochondrial genome. We also consulted the psychiatric team due to her delusional presentation. They attributed it to hyperammonemia and advised re-consulting if the delusions persisted after her ammonia levels were successfully managed. 

Unlike her previous admissions, despite an increased dose of sodium benzoate, the patient’s ammonia levels remained unsatisfactory on day 5. Her sodium benzoate dosage was then further increased to 3 g TDS, but by day 7, her ammonia levels were untouched. As per British Inherited Metabolic Disease Group (BIMDG) management guidelines, with the support of the metabolic team, intravenous ammonia scavengers, including arginine and sodium phenylbutyrate, were introduced while continuing oral sodium benzoate at 3 g TDS. 

**Figure 1:**
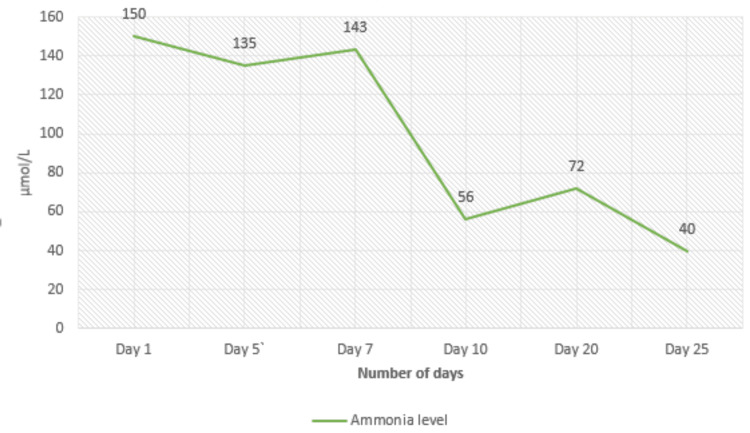
Trend of ammonia levels during admission

Considering her background of diabetes, she was administered 8.8 g of arginine and 22 g of sodium phenylbutyrate in 500 mL of 5% dextrose over 24 hours, although BIMDG suggests 10% glucose. She responded well, with ammonia levels stabilizing below 100 µmol/L. Clinically, her condition improved significantly, with her ataxia and confusion gradually resolving, returning her to baseline function. Over the following two weeks, she was transitioned to a combination of intravenous and oral therapy. Her IV doses were adjusted to 5 g of arginine and 10 g of sodium phenylbutyrate while maintaining oral arginine at 1 g QDS and glycerol phenylbutyrate at 3.3 g TDS. By the end of the third week, she was fully transitioned to oral ammonia scavengers: arginine 2 g QDS, sodium phenylbutyrate 6.6 g TDS, and sodium benzoate 3 g TDS. Since the patient remained at her functional baseline despite ammonia levels >50 but <100, reducing her ammonia to below 50 µmol/L proved challenging; the metabolic team recommended setting her target ammonia level at 100 µmol/L. The patient’s ammonia trend and plasma amino acid levels in response to treatment are noted below in Figure 1 and Table 3.

**Table 3:** Plasma amino acids level after IV ammonia scavengers

Plasma amino acid	On admission	After treatment	Normal range
Citrulline	66	48	10-48 umol/L
Arginine	111	143	12-145 umol/L
Taurine	55	108	28-212 umol/L
Valine	120	58	77-335 umol/L
Aspartic acid	12	10	4-52 umol/L
Cystine	7	22	10-80 umol/L
Hydroxyproline	16	30	6-75 umol/L
Methionine	42	34	11-43 umol/L
Threonine	168	197	47-228 umol/L
Isoleucine	39	12	27-101 umol/L
Serine	84	94	66-231 umol/L
Leucine	81	23	52-206 umol/L
Asparagine	44	51	23-80 umol/L
Tyrosine	146	103	32-125 umol/L
Glutamic acid	166	121	21-194 umol/L
Phenylalanine	93	83	35-103 umol/L
Glutamine	629	549	227-735 umol/L
Ornithine	92	153	24-144 umol/L
Proline	276	225	98-429 umol/L
Lysine	177	149	70-259 umol/L
Glycine	321	367	133-455 umol/L
Histidine	77	93	42-108 umol/L
Alanine	389	574	138-565 umol/L
Tryptophan	38	27	19-107 umol/L

At this stage, assistance from the psychiatric team was sought due to her persistent delusional beliefs. Based on their evaluation, she was diagnosed with erotomanic delusion and commenced on Amisulpride 200 mg BD. Gradually, her condition improved, and she expressed remorse for her actions. According to the psychiatric team, her newly diagnosed erotomania (De Clerambault's syndrome) may have been secondary to prolonged or recurrent hyperammonemia. She was subsequently discharged home on oral ammonia scavengers (arginine 2 g QDS, sodium phenylbutyrate 6.6 g TDS, and sodium benzoate 3 g TDS ) and scheduled for follow-up in the metabolic, gastroenterology, and psychiatry clinics in two weeks. 

After 10 days of discharge, the patient was brought to accident and emergency department with reduced responsiveness. Unfortunately, she shortly passed away before any further tests could be performed. According to her family, she was non-compliant with medications a few days prior to deconditioning.

## Discussion

The human body naturally produces around 17 g of ammonia daily, primarily from the breakdown of nitrogenous substances in the intestines and protein degradation during physical exertion [4]. Ammonia can cross the blood-brain barrier with ease, leading to neurotoxicity at elevated levels. The neurological symptoms associated with hyperammonemia can range widely in severity, from minor issues such as irritability, headaches, cyclical vomiting, and behavioral disturbances to more severe outcomes like intellectual disability, seizures, ataxia, and even coma. In adults with late-onset presentations, psychiatric symptoms, including manic episodes and psychosis, are also possible [5]. In pregnancy, complications from hyperammonemia may mimic more common issues, making it challenging to diagnose. Symptoms such as nausea, vomiting, headaches, mood disturbances, and seizures may be misattributed to hormonal changes, while postpartum mental status alterations could be confused with postpartum depression or psychosis [6]. The body employs a sophisticated system to neutralize ammonia toxicity. Ammonia binds temporarily to glutamine, a less toxic amino acid, for transport to the liver via the portal circulation. In hepatocytes, ammonia is transformed into urea, a water-soluble compound, through the urea cycle [7]. Liver cirrhosis is a well-known cause of elevated ammonia levels; however, non-cirrhotic hyperammonemia can be diagnostically complex. This condition arises from factors either increasing ammonia production or impeding its clearance, as outlined in Table 4 [7].

**Table 4:** Noncirrhotic causes of hyperammonemia UCDs, urea cycle disorders

Increased ammonia production	
Urea-producing bacterial infection	Escherichia coli, Klebsiella, Proteus mirabilis, Providencia rettgeri, Morganella morganii, Mycobacterium genavense, diphtheroids
Organ transplant	
Hemato-oncological disorders	Chemotherapy for acute leukemia, multiple myeloma, 5-fluorouracil, bone marrow transplantation
Protein load and increased catabolism	Steroid use, starvation, increased exercise, total parenteral nutrition, gastrointestinal bleeding
Decreased ammonia excretion	
Zinc deficiency	
Urerosigmoidostomy	
Drug induced	Valproic acid, glycine, carbamazepine, ribavirin, sulphadiazine with pyrimethamine, salicylate
Portosystemic shunts	Congenital intra/extrahepatic
Inborn errors of metabolism	Urea cycle disorders, defects in β-oxidation of fatty acids, organic acidemia, disorder of pyruvate metabolism

IEMs contributing to hyperammonemia include UCDs, organic acidurias, defects in fatty acid oxidation leading to carnitine deficiency, dibasic aminoaciduria, and pyruvate metabolism abnormalities [8]. The urea cycle consists of six enzymatic reactions, with three occurring in the mitochondria and the remaining three in the cytosol. Key enzymes, carbamoyl phosphate synthetase (CPS), ornithine transcarbamylase (OTC), arginosuccinate synthetase (ASS1), argininosuccinate lyase, and arginase, play crucial roles in this process [7]. Deficiencies in these enzymes have been linked to hyperammonemia due to disruption of the urea cycle [9,10]. Although the prevalence of UCDs is estimated at one in 30,000 live births, actual rates may be higher, as many cases remain undiagnosed or lead to early mortality [8].

**Figure 2:**
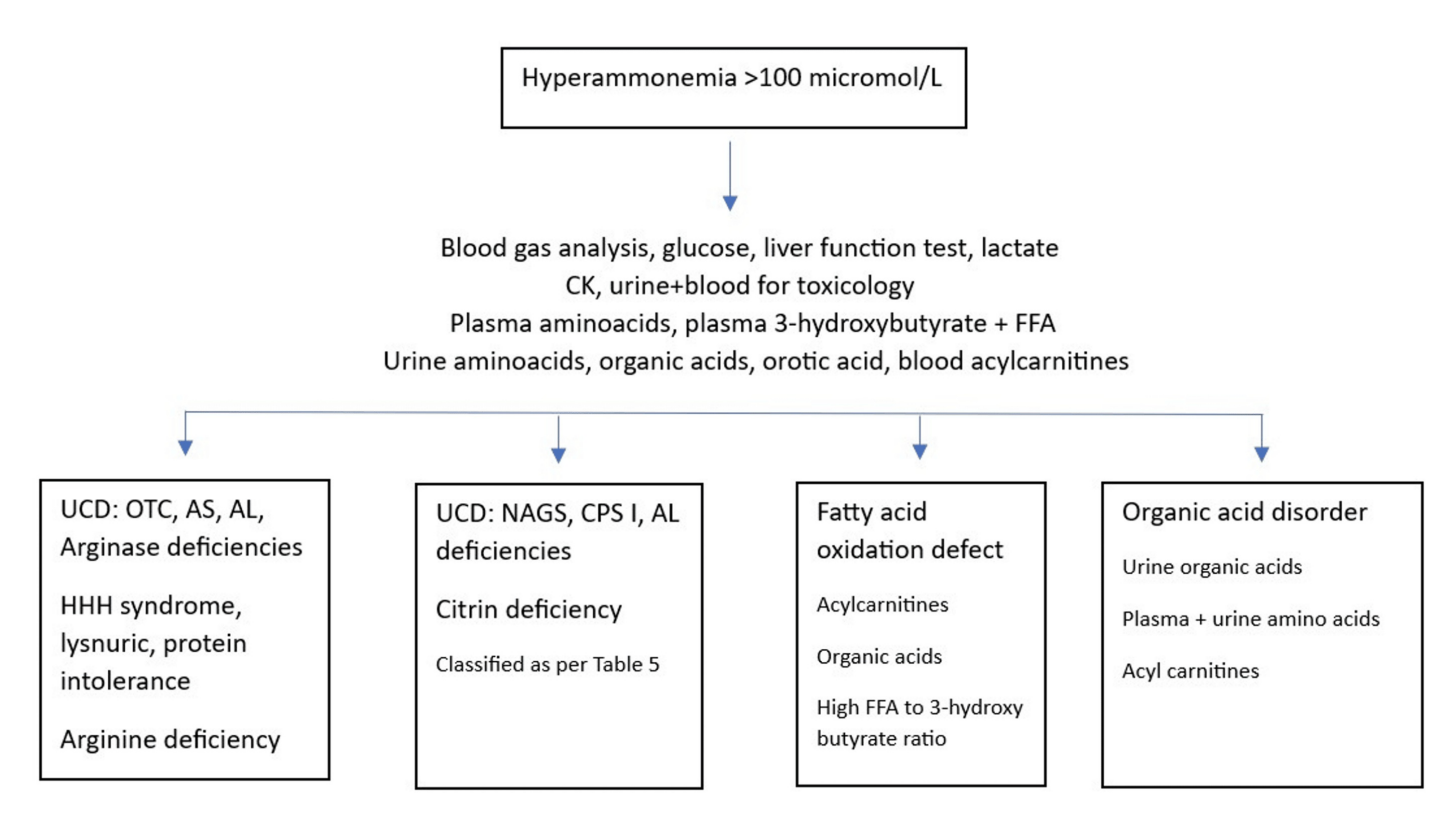
Approach to noncirrhotic hyperammonemia UCD: Urea cycle disorder, OTC: Ornithine transcarbamylase, AS: Arginosuccinic acid synthetase, AL: Argininosuccinic acid lyase, HHH: Hyperornithinemia-hyperammonemia-homocitrullinuria, NAGS: N-acetylglutamate synthetase, CPS I: Carbamyl phosphate synthetase I, FFA: Free fatty acid [11].

Diagnosing UCDs requires a comprehensive approach that includes clinical evaluations, biochemical tests, and molecular assessments. Essential tests include serum NH3, acid-base status, glucose, lactate, pyruvate, ketones, plasma amino acids, and urinary organic and orotic acid levels to identify specific urea cycle issues and rule out other IEMs. Figure 2 presents an overview of the diagnostic approach for non-cirrhotic hyperammonemia [8].

UCDs can be distinguished based on plasma and urine amino acid levels and urine orotic acid levels as shown in Table 5. 

**Table 5:** Plasma amino acid, urine amino acid, and urine orotic acid levels in each urea cycle enzyme defect OTC: ornithine transcarbamylase, ORNT I: mitochondrial ornithine transporter I, HHH: hyperornithinemia-hyperammonemia-homocitrullinuria

Defect	Plasma amino acid	Urine amino acid	Urine orotic acid
OTC	↓citrulline, ↓arginine	Nonspecific	↑
Carbamyl phosphate synthetase I	↓citrulline, ↓arginine	Nonspecific	N/↓
N-acetylglutamate synthetase	↓citrulline	Nonspecific	N/↓
Arginosuccinic acid synthetase citrullinemia I	↑↑citrulline, ↓arginine	↑↑citrulline	↑
Aspartate glutamate carrier citrullinemia II	↑citrulline, mildly ↑arginine, ↑threonine: serine ratio	Nonspecific	N
Argininosuccinic acid lyase: argininosuccinic aciduria	↑citrulline, argininosuccinic acid +	↑↑argininosuccinic acid, ↑citrulline	N/↑
Arginase I: argininemia	↑arginine	↑cystine, ornithine, arginine, lysine, glutamine	↑
ORNT I: HHH syndrome	↑ornithine	↑homocitrulline +/- ↑ornithine	N/↑
Cationic amino acid transporter: lysinuric protein intolerance	↓ornithine, arginine, lysine; Postprandial: ↑ glutamate, alanine	↑↑lysine, ↑ornithine, ↑arginine	Postprandial ↑

Confirmatory testing can involve enzyme analysis from fibroblasts or liver tissue and, if available, genetic mutation testing. Liver biopsies may be unnecessary if metabolite abnormalities provide a clear diagnosis or if a molecular diagnosis is feasible. Mutation analysis is preferred for definitive diagnosis of UCDs, as it can differentiate genetic causes from non-genetic factors, such as medication or nutritional deficiencies, which may impact prognosis [12,13].

The most prevalent genetic UCD is OTC deficiency, caused by mutations in the OTC gene on the X chromosome (Xp21.1) and inherited in an X-linked pattern [14]. Though usually presenting in neonates, partial OTC deficiency may lead to delayed symptoms in heterozygous individuals [15]. Diagnostic indicators include high urinary orotic acid, elevated glutamine, and low to normal citrulline levels [16].

Citrin deficiency (citrullinemia type II), stemming from mutations in the SLC25A13 gene encoding for citrin, impairs aspartate-glutamate exchange in the mitochondria of liver cells. Citrin plays a role in the malate-aspartate shuttle, critical for energy production and nucleotide synthesis. Citrin deficiency typically affects Western Asian populations, with patients often developing food aversions, especially to carbohydrates, preferring protein-rich diets as they age [17]. Symptoms tend to emerge later in life, generally between ages 11 and 79, and include delirium and behavioral changes, with some cases experiencing a gradual progression to liver cirrhosis [18].

In cases of acute hyperammonemia, prompt treatment is essential, even before a definitive diagnosis is available, ideally under guidance from a specialized metabolic center. In the UK, the BIMDG provides emergency treatment protocols for such cases [18]. The initial goals are to lower nitrogenous waste production and plasma ammonia levels to prevent cerebral edema. Hemodialysis can reduce plasma ammonia more quickly and effectively than hemofiltration or peritoneal dialysis though it may lead to a catabolic state by removing nutrients from the plasma [8]. During an acute decompensation episode, dietary management is vital to minimize catabolism and maintain nutritional balance, with calories provided through enteral or parenteral routes [19]. UCD dietary interventions aim to reduce nitrogen production by limiting protein intake and providing protein-free energy sources, often supplemented with nitrogen-scavenging medications [20].

Primary dietary interventions include tailored adjustments depending on the specific UCD and may involve arginine or citrulline supplementation. Alternative pathway therapies, like sodium benzoate, sodium phenylacetate (typically administered as sodium phenylbutyrate), and high-dose arginine, facilitate nitrogen excretion by generating metabolites readily excreted through the urine [8]. Glycerol phenylbutyrate is another option, preferred by some patients due to its better tolerance than sodium phenylbutyrate. When available, monitoring plasma levels of benzoate and phenylacetate can help manage potential toxicity, particularly in pregnancy, where the safety of phenylbutyrate remains uncertain, making sodium benzoate a safer alternative [8].

Liver transplantation offers a curative option for UCDs, particularly in patients with recurrent decompensation or who do not respond to conventional treatments. Though it represents a high-risk intervention, it may be considered a last resort following thorough risk-benefit discussions, especially for those experiencing neonatal onset or challenging late-onset UCDs [19].

## Conclusions

The causes of noncirrhotic hyperammonemia in patients can vary from straightforward conditions like a UTI or medication like valproate to more complicated ones like hemato-oncological illnesses, portosystemic shunts, or inborn metabolic abnormalities. It is important to know how to differentiate these based on simple biochemical tests and confirm them by mutation analysis if a genetic cause is suspected. This case underscores the challenges of recognizing non-cirrhotic hyperammonemia, particularly in the differential diagnosis of IEMs that may respond to ammonia-scavenging therapies administered empirically, either in the absence of or while awaiting a confirmed genetic diagnosis.
